# Personalizing digital mental health: a micro-randomized trial analysis of cognitive behavioral therapy/dialectical behavioral therapy text messages to improve mood

**DOI:** 10.1093/jamiaopen/ooag156

**Published:** 2026-10-13

**Authors:** Caroline A Figueroa, Jing Xu, Marvyn R Arévalo Avalos, Bibhas Chakraborty, Adrian Aguilera

**Affiliations:** Faculty of Technology, Policy, and Management, Delft Technical University, Delft, 2628, The Netherlands; School of Social Welfare, University of California Berkeley, Berkeley, CA, 94720, United States; Department of Statistics and Business Analytics, College of Business and Economics, United Arab Emirates University, Al Ain, Abu Dhabi, 15551, United Arab Emirates; Centre for Biomedical Data Science, Duke-NUS Medical School, National University of Singapore, Singapore, 169857, Singapore; School of Social Welfare, University of California Berkeley, Berkeley, CA, 94720, United States; Department of Psychiatry and Behavioral Sciences, University of California–San Francisco, San Francisco, CA, 94107, United States; Centre for Biomedical Data Science, Duke-NUS Medical School, National University of Singapore, Singapore, 169857, Singapore; School of Social Welfare, University of California Berkeley, Berkeley, CA, 94720, United States; Department of Psychiatry and Behavioral Sciences, University of California–San Francisco, San Francisco, CA, 94107, United States

**Keywords:** mental health, text-messaging, micro-randomized trial, cognitive behavioral therapy, digital health

## Abstract

**Purpose:**

Digital mental health interventions could improve population health. However, our understanding of how to improve their effectiveness through personalization is limited. We describe the outcomes of a micro-randomized trial (MRT) of a 60-day text messaging program, delivered during the final stages of the COVID-19 pandemic. We analyze the effects of 6 different types of cognitive behavioral therapy (CBT) and dialectical behavioral therapy (DBT) messages, and examine whether socio-demographic, health-related, and contextual factors (eg, timing of sending a message) moderate effectiveness.

**Methods:**

This secondary analysis included 373 adults aged 18 years or older (mean = 38.07), mostly English-speaking female, highly educated White individuals who reported being in good health, but having high stress and struggling to get their basic needs met. Participants were recruited between December 2021 and September 2022 through social media and a recruitment service (Health Match). We used the weighted and centered least squares method to examine the proximal effects of the text-message categories on daily mood, and whether sociodemographic, health-related factors, and the timing of message delivery moderated effectiveness.

**Results:**

None of the 6 CBT/DBT-message categories significantly improved mood ratings marginally. However, their effectiveness varied based on the time of day and self-reported health. Overall, sending a message in the afternoon was more effective. However, certain message categories showed significant effects when sent in the morning among participants with poorer self-reported health, while some showed lower effectiveness when sent in the late afternoon for participants with better health.

**Conclusion:**

Our analysis underscores the importance of considering the time of day and individual health status when developing digital mental health interventions. These insights can help in designing more personalized, adaptive, and effective digital interventions.

## Introduction

Mental health symptoms are common worldwide and increased significantly during the COVID-19 pandemic.^1^ The use of digital mental health interventions holds tremendous potential for improving population mental health by providing accessible and scalable support through personal digital devices.^2^^,^^3^ Text-messaging is a low-cost solution that is widely accepted among diverse populations, including those with lower incomes who may not own smartphones or utilize apps regularly.^4^ For instance, a systematic review showed that text messaging, particularly using cognitive behavioral therapy (CBT) and delivered more than twice per week, is effective in decreasing depressive symptoms in adults.^5^ However, low uptake, usage, and engagement with text-messaging interventions in real-world situations remain a significant concern.^4^ Our understanding of how these interventions can be most effective through personalization, such as delivering texts at optimal times with the right content, is still limited.^5^ These personalization factors may significantly influence the engagement and effectiveness of texting interventions.

Mobile phones can be used to deliver just-in-time adaptive interventions (JITAIs),^5–7^ which send push notifications, such as text messages, at moments when an individual is most receptive or in need, using Artificial Intelligence (AI) and algorithms. The micro-randomized trial (MRT) design^8^ tests the proximal effects (ie, the immediate, short-term impacts of intervention components on outcomes measured shortly after delivery) of the intervention categories to aid the design of JITAIs. In this study, we examine the proximal effect of receiving a text message on mood ratings collected 3 hours later. In an MRT, a participant is randomly assigned to one of the available intervention options (eg, message categories) at decision time points (eg, 4 possible times per day). Unlike traditional designs such as the randomized controlled trial (RCT), which evaluate an intervention as a whole, the MRT design allows researchers to understand the effects of intervention components, the average treatment effect of the intervention, when and for whom they are effective, and what factors moderate these effects. This paves the way for designing more personalized support.

During the COVID-19 pandemic, our research team developed a 60-day text messaging program called “Stay Well Intervention.”^9–11^ This low-intensity program, based on CBT and dialectical behavioral therapy (DBT), is designed to help individuals cope with the stress caused by COVID-19. The program is grounded in the idea that one’s thoughts and behaviors can determine how we feel and that engaging in “healthier” (more adaptive, balanced, and flexible) thoughts and behaviors can improve mood and reduce anxiety. CBT and DBT challenge unhelpful thoughts and help individuals understand the relationship between healthy behaviors and mood, showing positive effects in preventing^12^ and treating depression.^13^ CBT programs aimed at depression typically consist of multiple components, each with its own goals, content, and techniques.^14^^,^^15^ Two prior trials of The StayWell at Home program were evaluated, and both showed lower depression and anxiety among participants who completed the intervention.^10^^,^^16^ Further, an MRT study of these trials showed that StayWell participants improved their daily mood during the first 2-3 weeks of the intervention and were sustained during the rest of the study.^9^

Our research team recently launched a third phase of the StayWell program; this time expanding the reach of the intervention and the intervention content (eg, tip-message categories). Because different techniques may have varying effects on individuals,^17^ in Phase III we designed 6 categories of messages based on CBT and Dialectical Behavioral Therapy (DBT) skills, such as tips for identifying pleasurable activities, building mastery, and dealing with stress. As such, we aimed to choose categories representative of different clinical interventions and classify the messages into distinct categories. Here, we report the findings from Phase III only, conducted during the late phase and the end of the pandemic (December 2021-September 2022).

## Objective

This study aimed, for the first time, to gain insight into whether socio-demographic, health-related factors, and timing of the messages—all potential factors for personalization—influence the impact of different message categories. The main objectives of this paper are to estimate:

1. The proximal (ie, within 3-hour) effects of the text messages on mood ratings.
2. Whether the mood ratings are influenced by time (ie, days in the study), the selected time window on each day, and socio-demographic and health-related variables.
3. Whether sociodemographic, health-related, and time-related factors influence the effectiveness of the text messages on mood ratings.

This study will help identify knowledge for the optimization of intervention messages tailored to individuals’ characteristics and context (eg, developing just-in-time adaptive interventions), allowing for greater personalization and thereby effectiveness, leading to better mental health outcomes.^18^

## Study design

The design and procedures are also detailed in our main analysis paper (accepted for publication). The main outcomes paper evaluated the efficacy of the StayWell intervention across 3 intervention arms: Adaptive messaging, Random messaging, and Control arms. The results showed that StayWell participants (*n* = 1121) improved their mental health outcomes as evidenced by a 25% reduction in depression symptoms (PHQ-8) and 24% reduction in anxiety symptoms (GAD-7) following the 60-day intervention. The differences between the Adaptive arm of the Intervention, Random, and Control arms were not statistically significant. In the current study, we examine a subset of the full sample (*n* = 373, participants in the Random Arm only) and conducted an MRT rather than a pre/post efficacy trial. In addition, in this study we are evaluating mood ratings as opposed to the PHQ-8 and GAD-7 measures used in the main analysis. The MRT approach, instead of examining the effectiveness of the program as a whole, allows us to identify specific message components based that work best under different conditions. The institutional review board of the University of California (UC) Berkeley reviewed and approved all study procedures and the trial was pre-registered at ClinicalTrials.gov (NCT04473599).

### Participants

Participants were recruited via web-based ads on social media (Facebook, Instagram) and email invitations from HealthMatch Clinical Trials (Healthmatch.io). Facebook ads targeted English and Spanish speakers interested in mental health, linking to a Qualtrics baseline questionnaire. Ads were customized to target working-class adults aged 22 to 55 years, who were living in the United States and had an interest in mental health. HealthMatch recruited participants with higher symptom levels, prescreening and matching them to the study, then sending individualized email invitations. Eligibility criteria included US residency, age 18 or older, English or Spanish proficiency, completing a baseline questionnaire, having a US-based mobile phone, and not using internet-based phone numbers. Participants who completed the Qualtrics survey and passed phone verification were enrolled in the StayWell program via HealthySMS. Participants were excluded if they provided incomplete baseline data or registered with an internet-based phone number (eg, Google Voice) as these users were deemed to be associated with scam/spam accounts. The survey included validation questions and captchas to prevent fraud. Participants received US$20 upon study completion.

### Intervention

The StayWell Program is a 60-day text-messaging intervention designed to help participants learn mental health symptom management tips and monitor their mood via the delivery of daily text messages. The study included 3 intervention arms designed to test the efficacy of a reinforcement learning algorithm in personalizing the type and timing of mental health tip messages sent to users (Adaptive) vs sending tip messages at random (no personalization: Random) or not sending a tip message (Control). Randomization was conducted automatically within HealthySMS to ensure balanced allocation into the 3 intervention arms (block randomization 1:1:1), stratified by participant language. In this paper, we only examine the Random Arm, not the entire Phase III sample, to delve into the proximal effects of text messages. Compared to the earlier 2 phases, the third or final phase of the program contained 6 intervention messages and a no message, and the study was conducted during the late phase and end of the pandemic (December 2021-September 22). We reclassified, edited, or deleted past messages to more clearly fit into 1 of the 6 categories, removed references to social distancing and made the messages general to managing stress/mental health and not just coping with COVID, as was the case in the first 2 phases of StayWell. The final message bank had 192 messages with 6 distinct categories of CBT/DBT interventions and a “no message” category.

### Messages

Each day participants received 2 text messages, a randomly selected tip message (in English or Spanish) from the 7 categories (1 control, which is not sending any message, and 7 intervention messages, see Table 1) and sent daily at a random time between 9 am and 6 pm followed by a mood-monitoring message 3 hours later. In addition to the mental health tip message, all participants received a daily mood-rating message (“What is your mood right now from 1 to 9?”), and a mood-reminder message (“Please don’t forget to send in your mood rating”) delivered 50% of the time for all participants if they did not respond within 2 hours, and a mood-feedback message delivered either 33%, 66% or 99% of the time. Feedback messages reflected mood and provided general encouragement and/or reinforcement. We used the HIPAA-compliant platform, HealthySMS for the text-message delivery.

**Table 1.** Example messages per CBT/DBT category.

Category	Example message
**Accumulating positives (AP)**	Finding ways to play and laugh is super important for our mental and physical health.
**Building mastery (BM)**	For the next 5 days, take longer breaks from devices and social media.
**Distress tolerance (DT)**	It’s okay to take a break, lie down somewhere, or do nothing. Your body and mind deserve rest!
**Helpful thinking (HT)**	Every difficult situation also brings some positive things. Can you think of any positives for you?
**Interpersonal effectiveness (IE)**	A relationship is fruitful when it’s mutually giving. Listening is an essential skill that will invite a deeper connection.
**PLEASE skills (PS)**	Just 10 minutes of exercise can improve your mood and clear your mind. What’s something you can do today to be active?

### Micro-randomized trial design

The micro-randomized trials (MRT) are essentially sequential full factorial designs*—*that is, experimental designs where all possible combinations of all levels of each factor are included**.** At each decision time point, intervention options under each of the components are allocated randomly according to a full factorial design.^5^ Every day during the 60-day text-messaging study, treatment allocation was characterized by a full factorial design with a total of 2 factors or components representing messages (M) and the time frame (T) when the message was sent. M has 7 categories (no message (control), accumulating positives (ap), building mastery (bm), distress tolerance (dt), helpful thinking (ht), Physical, Eating, Avoiding Mood Altering Drugs, Balanced Sleep, Exercise, and Achieve Mastery (PLEASE) skills (ps), interpersonal effectiveness (ie)), and T has 3 levels (9 am-12 pm, 12 pm-3 pm, 3 pm-6 pm). We show examples of the messages in Table 1, with additional examples in Table S1. On each day within the MRT (see Figure 1), similar to the factorial designs, each participant was rerandomized to receive one message based on the combination of M (1/7 probability) and T (1/3 probability). Three hours following message delivery, participants received a message asking them to rate their mood on a scale of 1-9, with 9 being the most positive mood. We therefore define the proximal effect as the causal effect of receiving a message component on this subsequent mood rating measured 3 hours later.

**Figure 1.**
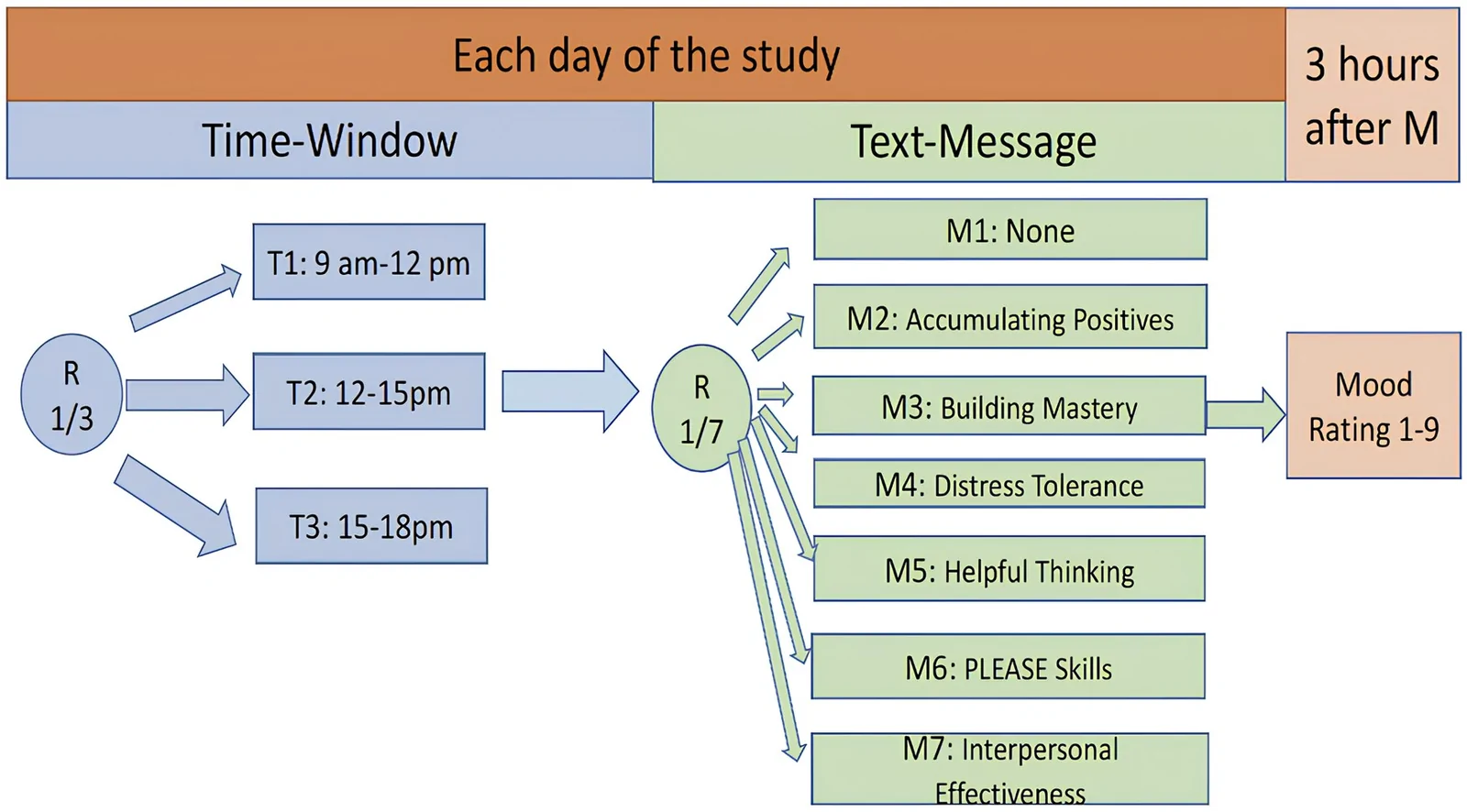
MRT design: R = randomization, M = message, T = time, study period = 60 days.

### Data and participants

All analyses in this paper pertain solely to the Random Arm. The Random Arm of the Phase III of StayWell at Home included 373 adults (note that the full study had 1121 participants) aged 18 years or older (mean = 38.07, SD = 10.60), mostly female (83.11%), with a mobile phone, speaking English (95.71%), with high education (57.1%), White (67.83%), high stress frequency (65.68%), good health (75.07%) and struggling to get their basic needs met (73.73%) (Tables 2 and 3). Almost half of the participants were currently employed.

**Table 2.** Continuous variables.

Variable	Mean (SD)
**Age**	38.07 (10.60)
**Baseline PHQ**	10.86 (5.71)
**Baseline GAD**	9.85 (5.44)
**Mood rating**	6.17 (1.92)

**Table 3.** Categorical variables.

Variable	*N* (%)
**Gender (female)**	310 (83.11)
**Education (college or above)**	213 (57.10)
**Employment (full time)**	185 (49.60)
**Ethnicity (White)**	253 (67.83)
**Stress (quite or more)**	245 (65.68)
**Health (good or great)**	280 (75.07)
**Language (English)**	357 (95.71)
**Basic need (somewhat hard)**	191 (51.21)
**Basic need (very hard)**	84 (22.52)

### Hypothesis

We have the following hypotheses based on the objectives listed in the introduction.

**Hypothesis 1** Intervention messages have marginal proximal effects on mood ratings compared to not sending a message, on average, across all time points and predictors.

**Hypothesis 2** The mood ratings are influenced by time (ie, days in the study), the selected time window on each day, and sociodemographic and health-related factors, including age, gender, education, employment, ethnicity, health status, and basic needs.

**Hypothesis 3** The proximal effects of intervention message categories are influenced by factors such as age, gender, education, employment, ethnicity, health status, basic needs, and the day or time window.

*Analysis of almost per protocol sample*: We reevaluated hypothesis 3 by including only participants with a minimum of 50 days (more than 80% of 60) of observations and the days with responding times of mood rating within the range of 3-5 hours. Note that 50-60 days are observed by the highest proportion of the participants (Figure S1).

### Statistical analysis

We operationalize proximal effects as the immediate impact of message delivery on mood ratings measured 3 hours later. All the above 4 hypotheses are tested using a weighted and centered least squares (WCLS) method.^19^ This method is similar to the generalized estimating equation (GEE) approach, which utilizes an independent correlation matrix with weights and centered intervention categories. It was specially developed for the causal effect estimation of MRTs (refer to the Supplementary Material for technical details). It provides robust proximal message effect estimates without introducing bias and includes covariates to reduce noise in the regression model. Another advantage of the WCLS method is the sequential randomization used to estimate the causal effect of the intervention messages. Power analyses can be conducted using the flexible MRT sample size calculator proposed by Xu et al.^20^ Though Boruvka et al’s (2018) method only considers components with binary categories (ie, control vs intervention), settings with multiple categories can be easily incorporated using Xu et al’s (2023) method.

## Results

### Effect of intervention messages on mood (hypothesis 1)

Table 4 presents the marginal proximal effect estimates of the intervention messages; see eqn (2) in the Supplementary Material for a formal definition of marginal proximal effects. We observe that, on average, within 3-5 hours after messages are sent, none of the 6 active message categories improve mood ratings by more than 0.06 (*P* > .3).

**Table 4.** Marginal text message effect on mood rating.

Category		Estimate	SE	*P* value
**Accumulating**	Positives	0.019	0.056	.739
**Building**	Mastery	0.031	0.059	.599
**Distress**	Tolerance	0.007	0.055	.9
**Helpful**	Thinking	0.052	0.054	.344
**Interpersonal**	Effectiveness	0.053	0.054	.332
**Please**	Skills	0.009	0.053	.869

### Hypothesis 2

To operationalize the influence of time on the mood ratings, 4 trends of the time (days in the study) are considered: constant, linear, quadratic, and linear plateau; see eqn (3) in the  Supplementary Material for further details. Table S2 presents the regression estimates of these trends, while Figure 2 displays plots of sample-averaged and regression-estimated mood ratings for each day within the study period. Based on lower *P* values, a simpler model, and closer alignment of regression-estimated mood ratings with the averages, we choose the linear plateau trend. Overall, mood ratings increase linearly over 2 weeks and then plateau at a maximum value.

**Figure 2.**
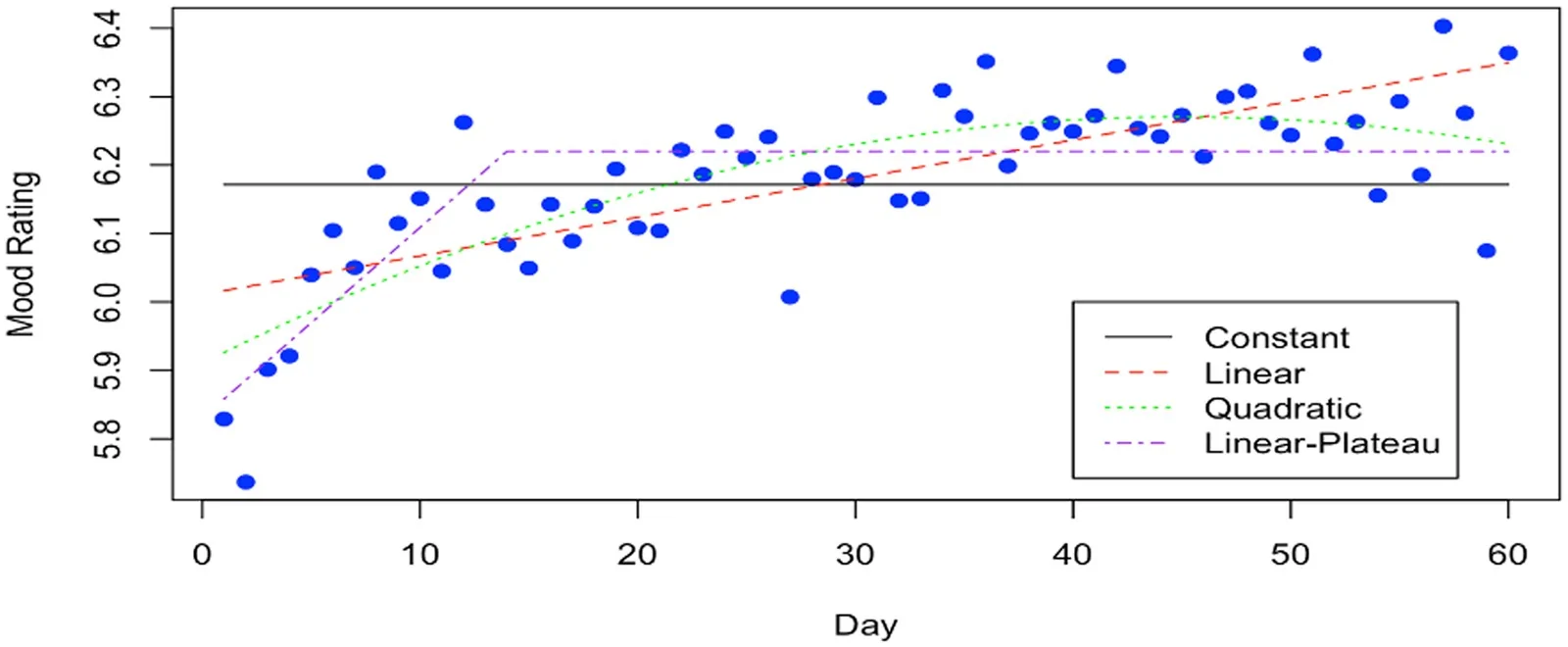
The sample-averaged (dots) and the regression-estimated (lines) based on the constant (solid), linear (dashed), quadratic (dotted) and linear plateau (dash dotted) trends for the mood ratings on each of the observed days.

Time of day was associated with mood: messages sent later in the day (3-6 pm) predicted higher mood ratings overall (Table S3, estimate = 0.086, *P* = .030), reflecting a main effect of time window rather than a moderation of treatment efficacy.

Furthermore, participants with greater self-reported health exhibit higher mood ratings overall, while individuals struggling with their basic needs report a lower mood rating. The findings are summarized in Table S3. See eqn (4) in the Supplementary Material for further details.

### Hypothesis 3

In this model, we added health status, basic needs, days in the study and time window, which are statistically significantly related to mood under hypothesis 2. In other words, these variables influence mood ratings in our analysis (see eqn (5) in the Supplementary Material for details). Table S4 presents not only the marginal proximal effect estimate of the intervention messages but also the proximal effect estimates conditioned on different values of the contextual variables. We observe that:

1. The proximal effects of building mastery (bm) and interpersonal effectiveness (ie) messages are positively related to mood (*P* < .010) when sent in the morning among participants with poor or fair health.
2. The proximal effect sizes are significantly lower when messages are sent in the late afternoon (3-6 pm) for the ap (*P* = .040) and PLEASE skills (*P* = .034) messages.
3. The proximal effect sizes are significantly lower among participants with good or better health for the bm (*P* = .005) and ie messages (*P* = .011).

### Analysis of almost per protocol

Table S5 presents the results repeating the analysis of Hypothesis 3 by considering only the participants with a minimum of 50-day observations and the days with responding times of mood rating within the range of 3 to 5 hours. We observe that:

1. The proximal effects of intervention message types are statistically significant (*P* < .05) when sent in the morning among participants with poor or fair health.
2. The proximal effect size is significantly lower when messages are sent in the late afternoon (3-6 pm) for the ap (*P* = .008) and ps (*P* = .013) messages.
3. The proximal effect sizes are significantly lower among participants with good or better health for the ap (*P* = .040), bm (*P* = .005), distress tolerance (dt) (*P* = .017) and ie (*P* = .001) messages.

In summary, none of the 6 active message categories showed significant effects in improving mood ratings on average versus sending no intervention message, however the effectiveness of intervention messages becomes apparent when considering certain contextual variables, such as health status, basic needs, and time of day. These findings remain qualitatively the same when we reanalyzed the data, only considering participants who were in the study for at least 50 days.

## Discussion

Here we report the outcomes of an MRT study with data from 373 individuals, who received supportive text messages during the final phase of the Stay Well program at the end of the COVID-19 pandemic. We show for the first time that the effectiveness of different types of intervention messages only becomes apparent when considering certain sociodemographic and contextual variables, such as how individuals self-report their health and the time of day at which the messages are sent. Thus, our analysis highlights the importance of considering the time of day, the health status of participants and the nuanced interaction between message type and these variables, when designing digital mental health interventions. These insights can help in designing more effective, personalized digital mental health interventions through text messaging by tailoring the timing and type of messages to the individual’s context.

### The effect of CBT/DBT messages on mood

Taken together, our findings suggest that while CBT and DBT techniques generally do not yield positive mood outcomes, the timing of message delivery and individual characteristics such as how one perceives their health may modulate the effectiveness of specific message types.

First, regardless of the message type, mood ratings were higher when sent in the afternoon compared to in the morning (during the 3-6 pm window). This is similar to results of our previous phases and is thus an important finding to replicate results in a new sample during the final phase of the COVID-19 pandemic. A potential explanation for these findings could lie in differences in the dynamics of mood regulation. Mood and emotional states can vary throughout the day, influenced by factors such as circadian rhythms, daily stressors, and health-related factors.^21–23^ For instance, a large-scale study of repeated measurements from the same participants across time over a 2-year period showed that mood is generally better in the morning.^21^ When messages are sent later in the day, their effect may therefore be more pronounced, leading to higher mood ratings after a message is sent.

Second, we find diverse effects of message categories on mood when we look at the interaction between timing, sociodemographic factors, and message category (different CBT/DBT techniques). For instance, the effect size of mood messages sent in the late afternoon was significantly lower for the accumulating positives and PLEASE skills (DBT) messages. One explanation is that these are more actionable messages (with suggestions for activities), which could be more helpful at the start of the day. Further, *building mastery* and *interpersonal effectiveness* messages had a positive effect when sent in the morning among participants with poor or fair self-reported health, but were less effective for participants with good or great self-reported health.

On potential explanation is that people with poor self-rated health, may have health problems that present more in the morning (eg, discomfort, fatigue, or the impact of health conditions on sleep quality). They may also need more motivation to engage in daily activities in a healthy way.

Furthermore, individuals may have different coping mechanisms or responses to CBT-based mood messages depending on health status,^24^ leading to differences in the effectiveness of message categories and their timings. Individuals with better health may already have greater coping skills,^25^ thus benefiting less from the CBT/DBT messages. In contrast to our hypothesis, we did not find any differences in mood ratings based on sociodemographic characteristics such as gender, income, and education.

Future research should explore daily dynamics and individual health differences in more detail to better understand their implications for mood-targeting texting interventions. Additionally, qualitative research on participant preferences could provide insights into why some components might have greater effects than others depending on health-related and contextual factors.

Because the timing text messages were sent, and the health status of participants influenced the proximal effects of messages on mood, our results provide evidence that the content of texting interventions could be tailored to health status and time of day for greater effectiveness. For example, the “building mastery” and “interpersonal effectiveness” messages may not be appropriate for participants who report good health as they may already be engaging in some of these approaches. However, these messages can be beneficial for participants reporting poor or fair health, especially if sent in the morning. Further, the “accumulating positives” and “PLEASE skills” messages might generally be less suitable for the late afternoon. This may be because each of these categories focuses on engaging in healthy behaviors and thus requires an element of planning ahead. If a message is received in the evening, there may be less opportunity to engage in one of these skills.

These findings are relevant for the development of JITAIs in several ways. This type of information could be used by reinforcement learning algorithms to learn from participant observations and adjust the timing of messages according to characteristics such as health status, including self-assessed health, and the presence of health conditions, optimizing mood outcomes. This information could be included at intake of the intervention, either from self-report or connecting with medical records. However, relying on these inputs may limit feasibility. Users may be unwilling or unable to provide self-reported data or grant access to health records, and JITAIs often depend on continuous data streams and reliable connectivity. These requirements may constrain their implementation, especially in low-resource settings.

Several studies have reported that AI-based messaging systems can personalize interventions and may be more effective for behavior change,^26^ though the research on this topic is still in the early stages.^27^ In addition, JITAIs Therefore, the results of this work could inform future reinforcement learning algorithms to optimize message generation.

### Limitations

We included a predominantly English-speaking, female, and White population, with a higher symptom severity, but in relatively good health. Our recruitment strategy through HealthMatch, a service that connects researchers to individuals seeking enrollment in clinical trials may have contributed to this, with a selection of individuals more motivated to use digital mental health tools. In the other 2 phases we recruited using targeted ads on social media and community-based sampling, leading to a more diverse sample. Taken together the resulting demographics may result in a participants that are not severely impacted and that are also familiar with mental health terminology. In addition, the study was conducted exclusively among US-based participants during a specific pandemic period and may not be generalizable to other populations and settings. Further, in this MRT analysis, we considered mood ratings as a continuous variable, we may have the limitation that the predicted proximal outcome does not guarantee to fall into the possible range of 1-9. However, in practice, no predicted values from our models fell outside the 1-9 scale, suggesting that treating mood as continuous did not produce clinically implausible predictions in this study.

### Future direction

In future work, we are evaluating whether we can extend the causal effect estimation methods for the messages to the binary or ordinal proximal outcomes. The novel methodologies are under development. Further, although we considered time of day, we did not examine the potential effects of the weekday, or season, which may also affect mood.^21^ This is an area for further research. Finally, future research should explore these dynamics and individual differences in more detail to better understand their implications for mood-targeting texting interventions.

## Conclusion

Our analysis suggests that the specific content of CBT/DBT-based messages only shows significant effects when considering the nuanced interaction between message type, timing, and individual context. In particular, the time the text messages are sent, and the health status of participants influence the proximal effects of messages on mood. This is an important finding for the optimization of text-messaging interventions for mental health and the development of JITAIs.

## Supplementary Material

ooag156_Supplementary_Data

## Data Availability

The data underlying this article will be shared on reasonable request to the corresponding author.
