# Supplementary material for: Personalizing digital mental health: a micro-randomized trial analysis of cognitive behavioral therapy/dialectical behavioral therapy text messages to improve mood
Source: JAMIA Open. 2026 Oct 13;9(5):ooag156. doi: 10.1093/jamiaopen/ooag156 (PMC13659918; doi:10.1093/jamiaopen/ooag156)
Supplement: Supplementary file 1 [file ooag156_supplementary_data.docx]

Supplementary material Figueroa et al.

**Supplementary table 1**

| **Code** | **Category** | **Messages** |
| --- | --- | --- |
| AP | Accumulating Positives | Finding ways to play and laugh is super important for our mental and physical health. |
|  |  | I know you might be feeling upset with how the current situation is playing out in your personal life. Be easy on yourself and try doing one thing a day that truly makes you happy. |
|  |  | Think of an activity you can do today that will make you feel better. What can you do to improve your mood? |
|  |  | What is one fun thing that you can do today? |
| BM | Building Mastery | For the next five days, take longer breaks from devices and social media. |
|  |  | How you speak to yourself matters! Take a couple minutes to practice some positive self-talk and think about things that you are proud. |
|  |  | Limiting your coronavirus media consumption to 10 minutes a day will keep you informed without making you needlessly worried or stressed. |
|  |  | If you need a moment, communicate it and take the moment. |
| DT | Distress Tolerance | Being willful (i.e., fighting reality) is a common response to stressful situations. What's one way you can practice willingness today? |
|  |  | It's okay to be distracted from work or household tasks during a world pandemic! You are doing the best you can. |
|  |  | It's okay to feel stressed during these hard times. You are evolving and growing every day. |
|  |  | It's okay to take a break, lie down somewhere, or do nothing. Your body and mind deserve rest! |
| HT | Helpful Thinking | Every difficult situation also brings some positive things. Can you think of any positives for you? |
|  |  | Fill in the blank. This situation sucks AND now I have time for …. |
|  |  | If you are feeling a bit sad or stressed right now, you are not alone. This is a hard time, but you can do this! |
|  |  | Is there something you miss from before the pandemic? Think of ways you can still incorporate that into your life. |
| IE | Interpersonal Effectiveness | A relationship is fruitful when it's mutually giving. Listening is an essential skill that will invite a deeper connection. |
|  |  | Be open to develop new relationships with people by connecting over shared values, interests, or experiences. |
|  |  | Being emotionally vulnerable with others can help us bond and remain calm. |
|  |  | Doing something as small as listening to a friend or family member can make a world of difference in their life. |
| PL | PLEASE Skills | Just 10 minutes of exercise can improve your mood and clear your mind. What's something you can do today to be active? |
|  |  | Setting a reminder to stop looking at screens one hour before bed time can help your bedtime routine. |
|  |  | Sleep is essential for your mood. Can you set a schedule for the time you go to sleep and get up in the morning? |
|  |  | Try to practice daily behaviors that prioritize your physical health, such as healthy eating, getting enough sleep, or exercise. |

**Details of MRT analysis**

At time *t*, let $A_{mt}$ denote the intervention of category-*m*; $Y_{t}$ denote the proximal outcome; ***H_t_*** be the history and ${\underline{\boldsymbol{X}}}_{\boldsymbol{t}}$ be the contextual variables (baseline characteristics or time dependent variables). Based on equation (5) of Boruvka et al (2018) and extension of binary intervention to multi-category intervention, we define the proximal effect of message *m* at time *t* by

$$E[E(Y_{t}|A_{1t}=0, \ldots,A_{mt}=0, \ldots,A_{Mt}=0, \boldsymbol{H}_{t}, I_{t}=1)-$$

$$E\left( {Y_{t}|A}_{1t}=0, \ldots,A_{mt}=0, \ldots,A_{Mt}=0, \boldsymbol{H}_{\boldsymbol{t}}, I_{t}=1 \right)|{S_{t}, I}_{t}=1]$$

$$={f_{mt}(S_{t})}^{T}\beta_{m}, (1)$$

where *I_t_* is the availability indicator (i.e., *I_t_* = 1), $\boldsymbol{H}_{\boldsymbol{t}}=({\underline{\boldsymbol{X}}}_{\boldsymbol{t}}\boldsymbol{,}{\underline{\boldsymbol{Y}}}_{\boldsymbol{t-1}}\boldsymbol{,}{\underline{\boldsymbol{A}}}_{\boldsymbol{t-1}})$, ***S_t_*** is a vector of summary variables chosen from ***H_t_***. The corresponding proximal outcome working model is

$E\left( {Y_{t}|A}_{t}, \boldsymbol{H}_{\boldsymbol{t}}, I_{t}=1 \right)={g(\boldsymbol{H}_{\boldsymbol{t}})}^{T}\boldsymbol{\alpha}+\sum_{m=1}^{M} [A_{mt}-\tilde{p}(A_{mt}=1|S_{t})]{f_{mt}(\boldsymbol{S}_{\boldsymbol{t}})}^{T}\beta_{m}$ (1)

where ${g(\boldsymbol{H}_{\boldsymbol{t}})}^{T}\boldsymbol{\alpha}$ is the working model of $E\left( {W_{t}Y_{t}\boldsymbol{|H}}_{\boldsymbol{t}} \right)$and $W_{t}$ is the weight, i.e., $W_{t}=\frac{\tilde{p}(A_{mt}|\boldsymbol{S}_{\boldsymbol{t}})}{p(A_{mt}|\boldsymbol{H}_{\boldsymbol{t}})}$. We have $p\left( \boldsymbol{H}_{\boldsymbol{t}} \right)=1/7$ (uniform randomization probability) and $\tilde{p}(A_{t}|\boldsymbol{S}_{\boldsymbol{t}})$ are estimated from the sample, i.e.,

- $\tilde{p}(A_{t}=None|\boldsymbol{S}_{\boldsymbol{t}})$**=**0.145,
- $\tilde{p}(A_{t}=Accumulating Positives|\boldsymbol{S}_{\boldsymbol{t}})$**=**0.140,
- $\tilde{p}(A_{t}=Building Mastery|\boldsymbol{S}_{\boldsymbol{t}})$**=**0.144,
- $\tilde{p}(A_{t}=Helpful Thinking|\boldsymbol{S}_{\boldsymbol{t}})$**=**0.138,
- $\tilde{p}(A_{t}=Distress Tolerance|\boldsymbol{S}_{\boldsymbol{t}})$**=**0.146,
- $\tilde{p}(A_{t}=Please Skills|\boldsymbol{S}_{\boldsymbol{t}})$**=**0.144,
- $\tilde{p}(A_{t}=Interpersonal Effectiveness|\boldsymbol{S}_{\boldsymbol{t}})$**=**0.143.

For the marginal text message effect estimation, we have

$E\left( Y_{t}|A_{t}, \boldsymbol{H}_{\boldsymbol{t}}, I_{t}=1 \right)={\boldsymbol{H}_{\boldsymbol{t}}}^{T}\boldsymbol{\alpha}+\sum_{m=1}^{M} [A_{mt}-\tilde{p}(A_{mt}=1|\boldsymbol{S}_{\boldsymbol{t}})]{\boldsymbol{S}_{\boldsymbol{t}}}^{T}\beta_{m},$	(2)

where $\boldsymbol{H}_{\boldsymbol{t}}$ and $\boldsymbol{S}_{\boldsymbol{t}}$ include only the intercepts, i.e., $\boldsymbol{H}_{\boldsymbol{t}}$**=**$\boldsymbol{S}_{\boldsymbol{t}}$=1.

For modelling the trajectory of mood rating, we have

$$E\left( {Y_{t}|\boldsymbol{H}}_{t}, I_{t}=1 \right)={\boldsymbol{H}_{t}}^{T}\boldsymbol{\alpha}, (3)$$

where $\boldsymbol{H}_{\boldsymbol{t}}$ includes intercept and ‘Day’ in study.

For the predictors of mood rating investigation, we have

$$E\left( {Y_{t}|\boldsymbol{H}}_{t}, I_{t}=1 \right)={\boldsymbol{H}_{\boldsymbol{t}}}^{T}\boldsymbol{\alpha}, (4)$$

where $H_{t}$ includes intercept, ‘Age’, ‘Gender’, ‘Education’, ‘Employment’, ‘Ethnicity’, ‘Health’, ‘basic needs’, ‘Day’ and ‘Time Window’.

For the causal text message effect estimation, we have

$$E\left( {Y_{t}|A}_{t}, \boldsymbol{H}_{t}, I_{t}=1 \right)={\boldsymbol{H}_{t}}^{T}\boldsymbol{\alpha}+\sum_{m=1}^{M} [A_{mt}-\tilde{p}(A_{mt}=1|\boldsymbol{S}_{\boldsymbol{t}})]{\boldsymbol{S}_{\boldsymbol{t}}}^{T}\beta_{m}, (5)$$

where $\boldsymbol{H}_{t}$ includes the intercept, ‘Health’, ‘basic needs’, ‘Day’ and ‘Time Window’ while $\boldsymbol{S}_{t}$ includes the intercept, ‘Health’ and ‘Time Window’.

An example of estimating the causal effect of the accumulating positive (ap) message from Table 6:

1. The marginal causal effect is

*E*(mood rate ∣ ap message) − *E*(mood rate ∣ no message) = 0.019.

2. The causal effect, conditioned on poor or fair health selected at the time window 9 am-12 pm, is

*E*[*E*(mood rate ∣ ap message) − *E*(mood rating ∣ no message) ∣ time window 9 am-12 pm and poor or fair health] = 0.278.

3. The causal effect, conditioned on good or better health selected at the time window 3 pm-6 pm, is

*E*[*E*(mood rate ∣ ap message) − *E*(mood rate ∣ no message) ∣ time window 3 pm-6 pm and good or better health] = 0.278–0.287–0.178 = −0.187.

**Supplementary Figure** 1: Frequency of the individual observing days

**Supplementary table 2**: Trajectory of Mood Rating

| Trend | Predictor | Estimate | SE | P-value |
| --- | --- | --- | --- | --- |
| Constant | Intercept | 6.172 | 0.073 | **0.000** |
| Linear | Intercept | 6.016 | 0.074 | 0.000 |
|  | Day | 0.006 | 0.002 | **0.001** |
| Quadratic | Intercept | 5.926 | 0.074 | 0.000 |
|  | Day | 0.016 | 0.004 | 0.000 |
|  | Day^2^ | 0.000 | 0.000 | **0.016** |
| Linear-Plateau | Intercept | 5.858 | 0.078 | 0.000 |
|  | Min(Day, 14) | 0.028 | 0.006 | **0.000** |

Supplementary table 3: Predictor of Mood Rating

| Predictor | Estimate | SE | P-value |
| --- | --- | --- | --- |
| Age | 0.001 | 0.007 | 0.865 |
| Gender(Female) | -0.165 | 0.213 | 0.437 |
| Education(College or above) | -0.069 | 0.150 | 0.645 |
| Employment(Full time) | -0.274 | 0.141 | 0.053 |
| Ethnicity(White) | -0.041 | 0.150 | 0.785 |
| Health(Good or better) | 0.476 | 0.184 | **0.010** |
| Language(English) | 0.008 | 0.576 | 0.989 |
| Basic need(Something hard) | -0.437 | 0.151 | **0.004** |
| Basic need(Very hard) | -1.088 | 0.232 | **0.000** |
| Min(Day, 14) | 0.027 | 0.006 | **0.000** |
| Time(12-15pm) | -0.005 | 0.034 | 0.891 |
| Time(15-18pm) | 0.086 | 0.039 | **0.030** |

**Supplementary Table 4:** Causal Effect of Text Messages on Mood Rating

| Message Category | Context | Estimate | SE | P-value |
| --- | --- | --- | --- | --- |
| Accumulating Positives | Marginal | 0.019 | 0.056 | 0.739 |
|  | Morning sent to poor/fair health | 0.278 | 0.151 | 0.065 |
|  | Time(12-15pm) | -0.081 | 0.131 | 0.539 |
|  | Time(15-18pm) | -0.287 | 0.140 | 0.040 |
|  | Health(Good or better) | -0.178 | 0.138 | 0.199 |
| Building Mastery | Marginal | 0.031 | 0.059 | 0.599 |
|  | Morning sent to poor/fair health | 0.383 | 0.142 | 0.007 |
|  | Time(12-15pm) | -0.084 | 0.129 | 0.513 |
|  | Time(15-18pm) | -0.083 | 0.134 | 0.534 |
|  | Health(Good or better) | -0.394 | 0.139 | 0.005 |
| Distress Tolerance | Marginal | 0.007 | 0.055 | 0.900 |
|  | Morning sent to poor/fair health | 0.232 | 0.137 | 0.089 |
|  | Time(12-15pm) | -0.013 | 0.131 | 0.921 |
|  | Time(15-18pm) | -0.187 | 0.139 | 0.179 |
|  | Health(Good or better) | -0.196 | 0.132 | 0.137 |
| Helpful Thinking | Marginal | 0.052 | 0.054 | 0.344 |
|  | Morning sent to poor/fair health | 0.176 | 0.148 | 0.233 |
|  | Time(12-15pm) | 0.040 | 0.128 | 0.753 |
|  | Time(15-18pm) | -0.143 | 0.139 | 0.303 |
|  | Health(Good or better) | -0.125 | 0.139 | 0.369 |
| Interpersonal Effectiveness | Marginal | 0.053 | 0.054 | 0.332 |
|  | Morning sent to poor/fair health | 0.408 | 0.147 | 0.006 |
|  | Time(12-15pm) | -0.126 | 0.128 | 0.325 |
|  | Time(15-18pm) | -0.209 | 0.135 | 0.122 |
|  | Health(Good or better) | -0.345 | 0.136 | 0.011 |
| Please Skills | Marginal | 0.009 | 0.053 | 0.869 |
|  | Morning sent to poor/fair health | 0.155 | 0.133 | 0.243 |
|  | Time(12-15pm) | -0.082 | 0.127 | 0.515 |
|  | Time(15-18pm) | -0.286 | 0.135 | 0.034 |
|  | Health(Good or better) | -0.050 | 0.123 | 0.682 |

**Supplementary Table 5**: Causal Effect of Text Messages based on the Participants with Minimum 50 Days Observation of Mood Rating and the Days with Responding Times of Mood Rating within the range of 3 to 5 hours

| Message Category | Context | Estimate | SE | P-value |
| --- | --- | --- | --- | --- |
| Accumulating Positives | Marginal | 0.030 | 0.066 | 0.652 |
|  | Morning sent to poor/fair health | 0.471 | 0.148 | **0.002** |
|  | Time(12-15pm) | -0.153 | 0.148 | 0.301 |
|  | Time(15-18pm) | -0.401 | 0.150 | **0.008** |
|  | Health(Good or better) | -0.309 | 0.150 | **0.040** |
| Building Mastery | Marginal | 0.065 | 0.077 | 0.393 |
|  | Morning sent to poor/fair health | 0.534 | 0.183 | **0.004** |
|  | Time(12-15pm) | -0.005 | 0.162 | 0.977 |
|  | Time(15-18pm) | -0.196 | 0.167 | 0.242 |
|  | Health(Good or better) | -0.520 | 0.184 | **0.005** |
| Distress Tolerance | Marginal | 0.014 | 0.066 | 0.830 |
|  | Morning sent to poor/fair health | 0.416 | 0.176 | **0.018** |
|  | Time(12-15pm) | -0.029 | 0.163 | 0.859 |
|  | Time(15-18pm) | -0.195 | 0.165 | 0.236 |
|  | Health(Good or better) | -0.401 | 0.168 | **0.017** |
| Helpful Thinking | Marginal | 0.119 | 0.073 | 0.101 |
|  | Morning sent to poor/fair health | 0.427 | 0.201 | **0.034** |
|  | Time(12-15pm) | -0.008 | 0.150 | 0.958 |
|  | Time(15-18pm) | -0.271 | 0.160 | 0.091 |
|  | Health(Good or better) | -0.281 | 0.193 | 0.146 |
| Interpersonal Effectiveness | Marginal | 0.054 | 0.071 | 0.449 |
|  | Morning sent to poor/fair health | 0.544 | 0.189 | **0.004** |
|  | Time(12-15pm) | -0.078 | 0.155 | 0.616 |
|  | Time(15-18pm) | -0.054 | 0.159 | 0.736 |
|  | Health(Good or better) | -0.578 | 0.179 | **0.001** |
| Please Skills | Marginal | 0.098 | 0.064 | 0.123 |
|  | Morning sent to poor/fair health | 0.449 | 0.164 | **0.006** |
|  | Time(12-15pm) | -0.280 | 0.147 | **0.056** |
|  | Time(15-18pm) | -0.377 | 0.151 | **0.013** |
|  | Health(Good or better) | -0.165 | 0.150 | 0.272 |
